# Endoscopic gas leak in diverted colon mimics perforation

**DOI:** 10.1093/gastro/goaf033

**Published:** 2025-04-24

**Authors:** Julia L Beilis, Bo Shen

**Affiliations:** Tony & Leona Campane Center for Excellence in Image-Guided Surgery & Advanced Imaging Research, Cleveland Clinic Cole Eye Institute, Cleveland, OH, USA; Center for Inflammatory Bowel Disease and the Global Center for Integrated Colorectal Surgery and IBD Interventional Endoscopy, Columbia University Irving Medical Center-New York Presbyterian Hospital, New York, NY, USA

## Introduction

Diversion colitis, or diversion proctitis, is an inflammatory disorder that occurs in portions of the colon and rectum following surgical construction of colostomy or ileostomy [1]. Although colostomies and ileostomies can significantly alleviate symptoms and discomfort associated with refractory inflammatory bowel disease (IBD) (such as perianal Crohn’s disease [CD]), diversion colitis can cause further discomfort and issues in patients who do experience symptoms [1, 2]. In symptomatic diversion colitis patients, symptoms typically include abdominal pain, pelvic pain or pressure, tenesmus, urgency, as well as bloody or mucus discharge [1]. However, it is unclear whether these symptoms are due to IBD or diversion colitis [2]. The inflammation that is seen in patients who are afflicted with diversion colitis is due to the increased sensitivity in the tissue that lines the colon. It is generally believed that friable mucosa of diversion colitis is caused by the lack of nutrients to colonic epithelium—particularly short-chain fatty acids (SCFAs) [3]. Long-term fecal diversion can also cause stricture in the distal large bowel or anorectal region. Patients with IBD and diverted large bowel still require periodic dysplasia surveillance [4]. Endoscopic evaluation of a diverted bowel can be challenging due to friable mucosa. Here, we present a case in which the patient develops a self-limited air leak from colonoscopy via the anus.

## Case report

A 38-year-old male patient was first diagnosed with CD in 2004 after experiencing bloody diarrhea. His initial colonoscopy was found to have ulcerations with multiple fistulous tracks, along with chronic ileocolitis. The patient was started on infliximab and mercaptopurine, and then underwent several hospitalizations for luminal disease and perianal abscess. After drainage of the abscess, a temporary diverting ileostomy was constructed, which was later reversed. The patient was then switched to adalimumab, but that medication was discontinued due to rashes. His disease course was further complicated after *Clostridioides difficile* infection, the development of multiple small and large bowel strictures, and recurrent perianal disease, which again required a diversion ileostomy in 2019. Following his diversion ileostomy, the patient developed a large parastomal hernia in 2021. In 2022, the patient was hospitalized for self-limited partial small bowel obstruction. Since December 2022, the patient has been on ustekinumab and budesonide.

**Figure 1.**
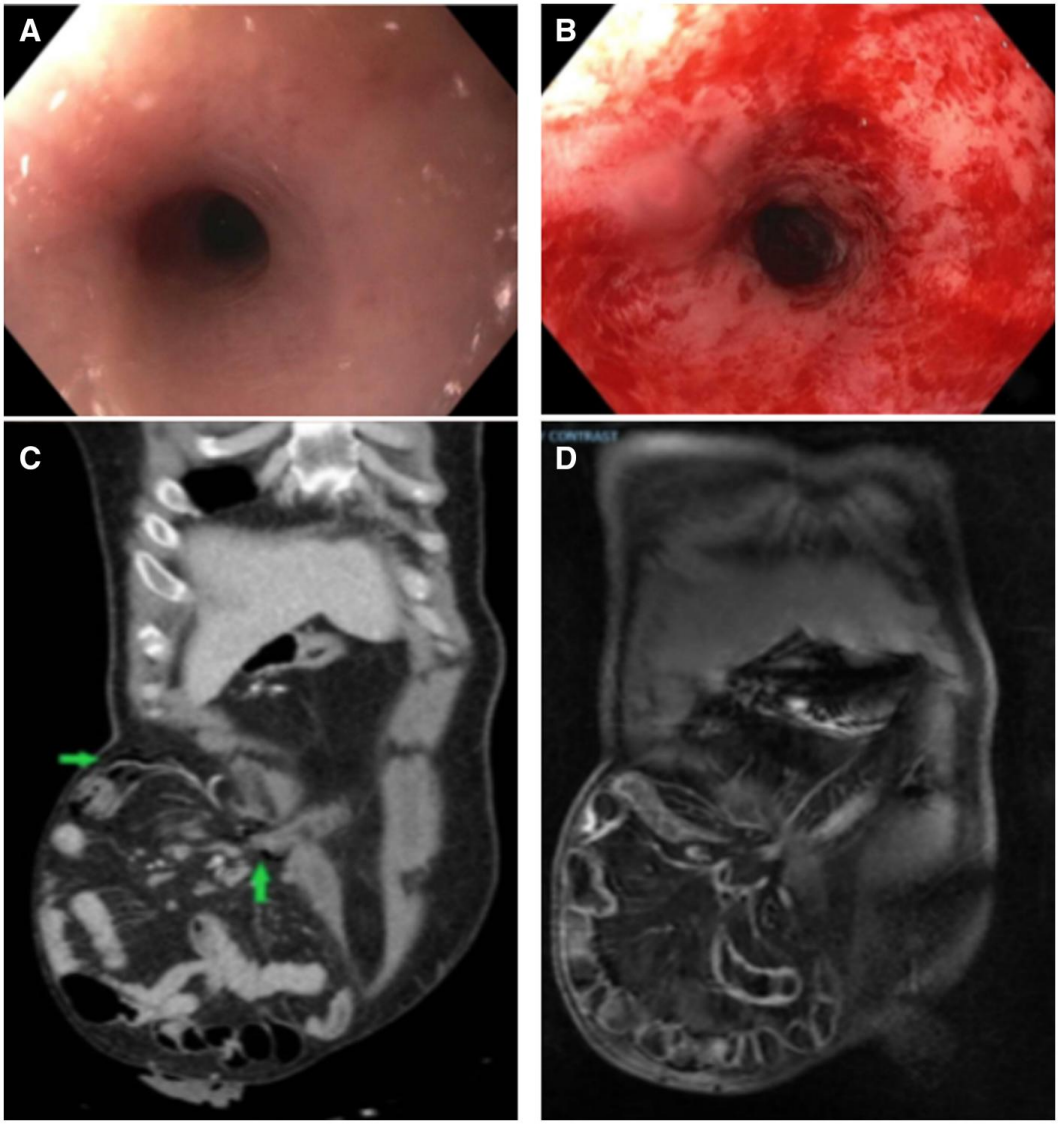
Endoscopic and imaging views of diversion colitis with air leak. (A) Mild anorectal ring stricture, traversable to a gastroscope. (B) Diversion colitis with friable mucosa. (C) Computed tomography following the ileoscopy and flexible sigmoidoscopy shows air leaks in the abdomen, as indicated by the arrows. (D) Magnetic resonance imaging after 1 week shows that previously seen air leaks in the abdomen have dissipated.

The patient was readmitted for abdominal pain. As part of the evaluation, ileoscopy via stoma and flexible sigmoidoscopy with carbon dioxide (CO_2_) were used. Ileoscopy showed no active mucosal inflammation. The same gastroscope was used to evaluate the diverted large bowel. Flexible sigmoidoscopy showed moderate diversion colitis with friable mucosa and loss of vascularity (Figure 1A). Anorectal biopsy was performed, which showed rectal mucosa with inactive chronic non-specific inflammation characterized by crypt distortion and full-thickness lamina propria lymph plasmacytosis with prominent lymphoid aggregates (Figure 1B). The patient developed mild bloating for which computed tomography was performed as a precaution. The computed tomography revealed a new pneumoperitoneum within the mesentery of the patient’s hernia sac that extended into the abdomen. The patient’s post-procedure symptoms resolved within 24 hours with no normal vital signs and white blood cell counts. It was believed that the patient had air leaks of CO_2_, rather than perforations, in the abdomen, which indicated that CO_2_ was pumped into the patient’s bowels during the flexible sigmoidoscopy (Figure 1C). This notion was verified by a follow-up magnetic resonance imaging of the abdomen and pelvis a week later, indicating that the gas leak into the abdomen was no longer present. No inflammatory change around the bowel was observed, or any fistulae or ascites (Figure 1D). The patient was scheduled to have elective surgery for the large ventral hernia repair.

## Discussion

Diversion colitis, as a result of loop or end colostomy or ileostomy, can compromise a patient’s quality of life if they are symptomatic. In diversion colitis, SCFAs can be used to provide nutrients to the colon epithelium [1]. While SCFA treatment for diversion colitis entails relieving symptoms, definitive therapy for diversion colitis is the re-establishment of bowel continuity by stoma closure [1, 2].

Some patients, as in this case, may not be candidates for stoma closure, for a variety of reasons. In those with underlying IBD, endoscopic diagnosis and surveillance of dysplasia of the diverted colon can be challenging, as the long-term diverted colon segment is inflamed and friable, and can spontaneously bleed, even due to gentle endoscopic gas insufflation. The senior author (B.S.) of this paper also noticed that transient bacteremia may occur following the endoscopic evaluation of a diverted large bowel. The current case verifies this notion in which the CO_2_ leaked through the friable mucosal layer of the diverted bowel.

Pumping of carbon dioxide into the diverted bowel, as seen in this case, resulted in an air leak into the patient’s peritoneal or retroperitoneal cavity, which is something that has yet to be discussed in medical literature. Previous literature has proposed a grading system for colonoscopic disruption of structural integrity in the wall of the colon [5]. However, in the case that we present, these gas leaks are not a true perforation or compromise of the wall structure of the large bowel. The high diffusion capability of CO_2_ along with the fragile epithelial tissue in the diverted colon is likely the cause of the gas leak. This case demonstrates how diversion colitis should be approached in clinical settings—the lack of nutrients, particularly SCFAs, to the diverted colon can contribute to CO_2_ leakage through the diverted colon. We want to alert the gastroenterology and endoscopy communities that transient, self-limited CO_2_ leakage is expected during endoscopy for long-term diverted bowel with inflammation. Prophylactic antibiotics may be used as a precaution.
